# Role of mitochondrial quality control in the pathogenesis of nonalcoholic fatty liver disease

**DOI:** 10.18632/aging.102972

**Published:** 2020-03-26

**Authors:** Ruibing Li, Sam Toan, Hao Zhou

**Affiliations:** 1 Department of Clinical Laboratory Medicine, The First Medical Center, Chinese PLA General Hospital, Beijing 100853, China; 2 Department of Chemical Engineering, University of Minnesota-Duluth, Duluth, MN 55812, USA; 3 Medical School of Chinese PLA, Chinese PLA General Hospital, Beijing 100853, China

**Keywords:** NAFLD, mitochondrial quality control, fission, fusion, mitophagy

## Abstract

Nutrient oversupply and mitochondrial dysfunction play central roles in nonalcoholic fatty liver disease (NAFLD). The mitochondria are the major sites of β-oxidation, a catabolic process by which fatty acids are broken down. The mitochondrial quality control (MQC) system includes mitochondrial fission, fusion, mitophagy and mitochondrial redox regulation, and is essential for the maintenance of the functionality and structural integrity of the mitochondria. Excessive and uncontrolled production of reactive oxygen species (ROS) in the mitochondria damages mitochondrial components, including membranes, proteins and mitochondrial DNA (mtDNA), and triggers the mitochondrial pathway of apoptosis. The functionality of some damaged mitochondria can be restored by fusion with normally functioning mitochondria, but when severely damaged, mitochondria are segregated from the remaining functional mitochondrial network through fission and are eventually degraded via mitochondrial autophagy, also called as mitophagy. In this review, we describe the functions and mechanisms of mitochondrial fission, fusion, oxidative stress and mitophagy in the development and progression of NAFLD.

## INTRODUCTION

The continuous intake of excess dietary fat without consumption of excessive alcohol is one of the main causes of non-alcoholic fatty liver disease (NAFLD), which includes a spectrum of liver pathologies such as steatosis, steatohepatitis, fibrosis and cirrhosis. Non-alcoholic steatohepatitis (NASH) is a liver disease that resembles the histology of alcoholic hepatitis, but occurs without the consumption of excessive alcohol. It represents one of the stages of NAFLD. More than 83.1 million people in the United States are diagnosed with NAFLD and 27% of these patients exhibit symptoms of NASH [1]. About 2%–3% subset of patients with NAFLD develop NASH, and 5%–8% of NASH patients develop liver cirrhosis within five years [2].

**Figure 1:**
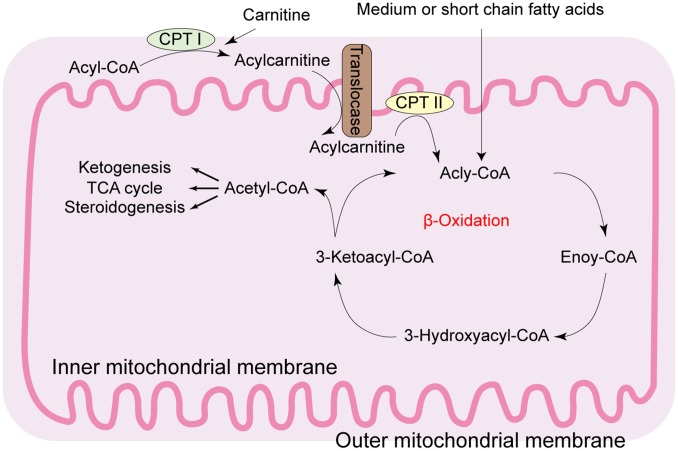
**Diagrammatic representation of the mitochondrial fatty acid β-oxidation.** During β-oxidation in the mitochondria, free fatty acids (FFAs) undergo step-wise enzymatic dehydrogenation, hydration, a second dehydrogenation, and thiolysis to generate a single 2-carbon acetyl-CoA molecule and a shortened fatty acid. The cycle is repeated until the fatty acid is completely broken down into its constituent acetyl-CoA subunits. The acetyl-CoA molecules enter the citric acid cycle to produce energy-rich NADH and FADH_2_ molecules that are then converted to ATP in the electron transport chain. Under fasting conditions, acetyl-CoA molecules are converted into ketone bodies (acetoacetate and β-hydroxybutyrate), which are released by the liver to be oxidized in peripheral tissues by the tricarboxylic acid cycle. CPT: carnitine palmitoyl transferase; TCA: tricarboxylic acid.

Hepatic steatosis occurs because of imbalance between hepatic lipid uptake, *de novo* lipogenesis, and lipid clearance [3]. As shown in Figure 1, lipid metabolism is primarily regulated by the mitochondria, which are enriched in the liver parenchyma cells [4]. Mitochondria are the powerhouse of eukaryotic cells, producing 90% of the cellular energy through oxidative phosphorylation (OXPHOS) in the form of adenosine triphosphate (ATP). Mitochondria are also major sites of reactive oxygen species (ROS) and regulate oxidative programmed cell death or apoptosis. Mitochondrial dysfunction in the hepatocytes is frequently associated with metabolic disturbances observed in fatty liver diseases. For example, chronic lipid consumption alters the status of mitochondrial oxidative phosphorylation in the hepatocytes by suppressing the activity and expression of OXPHOS complex proteins in the mitochondria. In NAFLD, the function and structure of the hepatocyte mitochondria is significantly altered.

**Figure 2:**
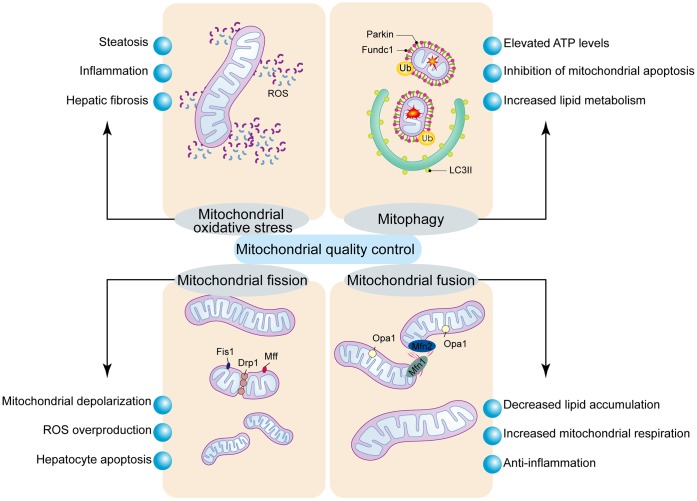
**Regulatory mechanism of mitochondrial quality control.** Mitochondrial oxidative stress induces mitochondrial dysfunction and hepatocyte apoptosis. Mitochondrial fission is modulated by Drp1 and its receptors, Fis1 and Mff. Excessive mitochondrial fission induces mPTP opening and mitochondrial dysfunction, which results in the activation of mitochondrial pathway of cellular apoptosis. Mitochondrial fusion is regulated by Mfn1/2 and Opa1, and stabilizes the mitochondrial membrane potential and blocks the mitochondrial pathway of apoptosis. Mitophagy is a process that breaks down damaged mitochondria and is controlled by Parkin or Fundc1.

The mitochondrial quality control (MQC) mechanism involves intricate regulation of several processes such as proteostasis, biogenesis, dynamics, and mitophagy, all of which are integral to maintaining cellular homeostasis as shown in Figure 2 [5–7]. The failure of the quality control processes results in mitochondrial dysfunction and is one of the underlying causes for NAFLD [8]. MQC involves coordinated regulation of a hierarchical network of pathways that act sequentially from an individual protein molecule to the whole organelle [9–11]. Antioxidant systems are the primary line of defense to maintain a functional mitochondrial redox environment and prevent oxidative damage [12, 13]. Mitochondria are highly dynamic organelles that constantly undergo fusion and fission, which are critical for mitochondrial homeostasis, mtDNA inheritance and intracellular distribution of the mitochondria [14–17]. Tight regulation of mitochondrial fission and fusion is required to constantly adapt to altering physiological needs [18–20]. When individual mitochondria or their constituents such as OXPHOS protein complexes and lipids are irreversibly damaged, they are degraded through mitophagy, a lysosome-dependent proteolytic system in order to maintain cellular homeostasis [21–24]. When sustained oxidative insults overwhelm the MQC mechanisms, it will result in significant mitochondrial injury that will detrimental to the function and survival of the hepatocytes. Preclinical evidence suggests that modulation of MQC is therapeutically beneficial against NAFLD/NASH [25–27]. In this review, we discuss recent studies regarding the involvement of MQC processes including mitochondrial fission, mitochondrial fusion, mitophagy, and mitochondrial oxidative stress in fatty liver diseases such as NAFLD and NASH. Elucidation of the molecular mechanisms underlying the defective MQC mechanisms is critical for the design of effective therapeutic strategies to prevent or cure fatty liver diseases.

## Oxidative Stress

Several anti-oxidative factors such as glutathione (GSH), superoxide dismutase (SOD) and glutathione peroxidase (GPx) regulate the levels of ROS that are mainly generated by the mitochondrial respiratory chain [28–31]. In the cytosol, enzymes such as amino acid oxidases, cyclooxygenases, lipoxygenases, nitric oxide (NO) synthase, and xanthine oxidase also generate ROS such as superoxide anions, peroxides and others [32–34]. ROS modulate several specific signaling pathways that regulate several cellular functions as well as pathological mechanisms in several human diseases [35–38]. The cyclooxygenases and lipoxygenases generate superoxide anions that drive arachidonic acid metabolism and inflammation, which play an important role in several cancers, whereas, the xanthine oxidase is implicated in oxidative stress during ischemia/reperfusion injury [39–41]. The oxidants generated by the sulfhydryl oxidases in the endoplasmic reticulum (ER) are required for disulfide bond formation, protein folding, and the assembly and secretion of proteins through the secretory pathway. Several peroxisomal oxidases generate hydrogen peroxide during the oxidative activities in the peroxisomes [42–44]. NADPH oxidases, the main source of ROS in several liver diseases, generate superoxide anions in the mitochondria by transferring a single electron from NADPH to molecular oxygen [45–47].

Emerging evidence suggests that the main site of superoxide generation in the mitochondria is the flavin mononucleotide group of complex I through reverse electron transfer, consistent with data that shows inhibition of succinate-related ROS generation by diphenyleneiodonium without affecting the flavin group of complex II [48–50]. Moreover, complex III of the mitochondrial respiratory chain generates ROS species through the ubiquinone-reactive sites, Q0 and Qi [51, 52]. The redox activity of 66-kDa Src homology 2 domain-containing protein (p66Shc) within mitochondria has been shown to directly generate hydrogen peroxide through oxidation of cytochrome c without intermediate formation of superoxide anion [53, 54]. The apoptosis-inducing factor (AIF) demonstrates NADH oxidase activity and generates superoxide and hydrogen peroxide [40, 55].

The high-fat diet-induced NAFLD is characterized by increased intracellular lipid accumulation, high mitochondrial ROS and hepatocyte apoptosis as shown in Figure 2 [56]. Mitochondrial oxidative stress also promotes lipid peroxidation in hepatocytes and enhances expression of hepatic CC-chemokines, which promote infiltration of CCR5-positive cells and activation of myofibroblasts resulting in extensive liver fibrosis [57]. The mitochondria in NAFLD/NASH patients exhibit increased rate of proton leakage because of upregulation and activity of the uncoupling protein-2 or UCP2 [58]. Moreover, altered activity of the mitochondrial ATP-sensitive potassium channels (mitoKATP) is associated with higher respiration rate and increased ROS generation [59]. Furthermore, in NAFLD patients, elevated mitochondrial permeability transition pore (mPTP) opening contributes to mitochondrial oxidative stress and apoptosis of hepatocytes [60]. These data suggest that mitochondrial oxidative stress plays an important role in hepatocyte apoptosis, steatosis, liver fibrosis, hepatic inflammation, and other pathophysiological changes that are involved in the development and progression of NAFLD.

Attenuation of mitochondrial oxidative stress is a promising strategy to reduce mitochondrial damage and slow the progression of NAFLD. Administration of mitoquinone mesylate (MitoQ), a mitochondrial-targeted antioxidant with high bioavailability, restores hepatic mitochondrial functions and ameliorates glucose intolerance and hepatic steatosis [61–63]. Antioxidant foods including blueberry juice and probiotics significantly reduce NAFLD-induced mitochondrial swelling and hepatic necrosis by restoring the mitochondrial respiratory chain function and suppressing the production of ROS [64]. Pretreatment with methionine alleviates the pathological changes associated with NAFLD by increasing GSH levels [60].

Several signaling pathways are related to mitochondrial oxidative stress and are potential targets to restoring mitochondrial respiratory functions [65–67]. Pharmacological activation of AMP-activated protein kinase α2 (AMPKα2) attenuates mitochondrial oxidative stress in NAFLD by increasing mitochondrial biogenesis and lipid oxidation [68]. Activation of the c-Jun N-terminal kinase-1 (JNK1) contributes to increased mitochondrial oxidative stress (as assessed by the GSSG:GSH ratio) and decreased ATP levels, which trigger mPTP opening and hepatocyte apoptosis [69]. Aberrant regulation of the Sirt1/STAT3 signaling pathway promotes mitochondrial damage and elevated production of ROS in NAFLD [70]. The noncanonical KEAP1-NFE2L2 pathway is a potential therapeutic target in NAFLD because it promotes antioxidative response against lipotoxicity in the hepatocytes and the mouse liver tissues [71]. Mitochondrial ROS production is also regulated by the Ras/Erk signaling pathway in NAFLD [72]. Thus, results from mouse models of mutant mitochondrial OXPHOS-related genes and pharmacological targeting of β-oxidation reveal that modulation of mitochondrial ROS and fatty acid oxidation can prevent metabolic dysfunction and hepatic pathology in NAFLD.

## Mitochondrial Fission

Mitochondria are dynamic organelles that are constantly changing their structure and shape through fusion and fission processes in response to changes in energy demand and supply [73, 74]. As shown in Figure 2, the changes in mitochondrial dynamics are associated with cell viability, apoptosis, and bioenergetic adaptations [75, 76]. Mitochondrial fission is observed when mitochondria are subjected to oxidative stress-induced damage and segregates damaged mitochondria from the normal ones [77–80]. The mitochondrial fusion- fission balance is disrupted by intracellular and extracellular stress, and the fragmented mitochondria form small spheres or short rods in comparison to extensive elongated network of the normal mitochondria [20, 81]. During the last decade, we have gained significant insights into the molecular basis of mitochondrial dynamics in relation to several biological processes such as apoptosis, autophagy, metabolism, development, and aging [82–84]. The molecular machinery governing mitochondrial dynamics was initially discovered in budding yeasts and Drosophila [85]. Subsequently, mammalian orthologs of key proteins involved in mitochondrial fission such as mitochondrial fission 1 (Fis1), dynamin-related protein 1 (Drp1), mitochondrial fission factor (Mff), mitochondrial dynamics proteins of 49 kDa and 51 kDa (MiD49 and MiD51) were discovered [86–89].

Activated Drp1 forms spiral-like structures upon oligomerization at the fission sites of the outer mitochondrial membrane (OMM) after being recruited from the cytosol and returns back to the cytosol upon completion of the fission process [20, 90–93]. Drp1 lacks the lipid-binding pleckstrin homology domain and interacts with other mitochondrial-resident proteins to drive the fission process [94]. Recent reports suggest that proteins such as Mff, Fis1, Mid49, and Mid51 recruit Drp1 to the outer mitochondrial membrane [95–97]. Fis1 is distributed throughout the outer membrane, whereas, Mff and MiDs demonstrate punctate organization along the mitochondrial tubules and exhibit stronger interactions with Drp1 compared to Fis1 [98, 99]. Fis1 and Mff independently contribute to Drp1 recruitment and oligomerization at the outer mitochondrial membrane, with Mff playing a more predominant role [100]. Furthermore, MiD49 and MiD51 can recruit Drp1 to the mitochondria in the absence of Mff and Fis1 [101, 102]. Dysregulation of the proteins involved in mitochondrial fission significantly alter the mitochondrial morphology and impair mitochondrial function [103, 104].

The levels of Fis1 and Drp1 proteins are reduced in the NASH model mice fed with Western diet for more than 2 months and accompanied by hepatic inflammation and liver fibrosis [105]. Overexpression of isocitrate dehydrogenase 2 (IDH2) reduces Drp1 and Fis1 levels in the hepatocytes and prevents NASH progression [106]. High-fat diet induces Drp1-related mitochondrial fission and reduces the levels of anti-inflammatory cytokines such as interleukin-10 (IL-10) and IL-13, thereby suggesting a role for mitochondrial fission in hepatic inflammation [107]. Decreasing mitochondrial fission alters the expression of genes involved in lipid metabolism and alleviates hepatic steatosis in a murine model of NAFLD [108]. Moreover, uncontrolled mitochondrial fission triggers hepatic fibrosis and liver inflammation resulting in increased hepatocyte death through the caspase-9-related apoptotic pathway [109]. Therefore, decreasing mitochondrial fission represents a novel therapeutic target for NAFLD.

Drp1-related mitochondrial fission is regulated by multiple mechanisms [110–113]. In NAFLD, p53 induces Drp1-related mitochondrial fission and mitophagy arrest, which results in mitochondrial dysfunction through mPTP opening, reduced mitochondrial potential, oxidative stress, calcium overload, and ATP depletion [109]. Besides, Drp1 transcript levels are regulated by the SIRT1/SIRT3-FOXO3a signaling pathway [114]. Moreover, nutrient stimuli promote post-transcriptional phosphorylation of Drp1 at Ser616 in the hepatocytes [115].

Several drugs regulate the activity of Drp1-related mitochondrial fission, but, their efficacy for the treatment of NAFLD remains to be established. Pharmacological doses of a first-line diabetic drug, metformin, activates the AMPK signaling pathway and promotes mitochondrial fission in the HFD-fed mice resulting in increased mitochondrial respiration, normalized mitochondrial membrane potential and upregulated ATP levels [116]. Mdivi-1, an inhibitor of Drp1-related mitochondrial fission, promotes apoptosis of hepatocytes by inhibiting mitochondrial depolarization and increasing ROS levels [117, 118]. Irisin is a newly discovered hormone secreted by muscle tissue that blocks mitochondrial fission and alleviates liver ischemia-reperfusion injury [119]. Resolvin D1 (RvD1), a specialized pro-resolving lipid mediator with anti-inflammatory and antioxidant activities, protects the liver against ischemia-reperfusion injury by suppressing Drp1-related mitochondrial fission [120].

## Mitochondril Fusion

Mitochondrial fusion in mammalian cells is mediated primarily by mitofusin-1 or Mfn1 and mitofusin-2 or Mfn2 [16, 121, 122]. Mfn1 proteins tether two opposing mitochondria through their individual HR2 domains [123, 124]. Mfn2 proteins oligomerize with either other Mfn2 proteins or with Mfn1 proteins to promote mitochondrial fusion. Moreover, Mfn2 promotes physical interactions between the ER and the mitochondria, which is essential for Ca^2+^ signaling [18, 125, 126]. The fusion of the inner mitochondrial membrane (IMM) is regulated by optic atrophy 1 (Opa1), which plays a critical role in maintaining the balance between mitochondrial fusion and fission [121, 127]. Opa1 is processed by the mitochondrial-processing peptidase (MPP) to generate a membrane bound long isoform (L-Opa1), which is further cleaved into the short isoform (S-Opa1) by the IMM peptidases such as the yeast mitochondrial DNA escape 1-like (YME1L) protease and the m-AAA protease, OMA1 [128, 129]. The IMM-bound L-Opa1 is required for fusion, whereas, the stress-activated soluble S-Opa1 limits fusion and promotes fission [130]. The levels of L-Opa1 and S-Opa1 are regulated by YME1L and OMA1 concentrations under basal conditions [131, 132]. The inhibition of mitochondrial fusion in the mouse embryonic fibroblasts through the suppression of Mfn1 and Mfn2 gene expression induces severe growth defects accompanied by changes in the mitochondrial membrane potential and decreased respiration [133, 134].

Fusion allows damaged mitochondria with oxidized lipids, proteins, and mutant mitochondrial DNA, and aberrant mitochondrial membrane potential to mix with healthy ones. This helps restore mitochondrial functions and maintain cellular homeostasis. However, mitochondria that suffer significant loss of membrane potential do not fuse and are subsequently degraded by mitophagy [135–138]. Moderate fusion prevents autophagy of mitochondria during nutrient starvation and excessive fusion of mitochondria inhibits mitophagy [139, 140]. Therefore, fusion of mitochondria is a compensatory and protective mechanism to fix the functional defects in some portions of the mitochondria in the mammalian cells. Metabolic changes and the status of energy supply determine the rate of mitochondrial fusion in the mouse embryonic fibroblasts [141].

Mfn1 expression is decreased in the hepatocytes in response to a high-fat diet and correlates with steatohepatitis [106]. The pro-inflammatory CXCR3 protein induces mitochondrial dysfunction in the NASH model mice by decreasing Mfn1 protein levels [142]. Moreover, reduced Mfn2 levels are observed in the liver biopsies from patients with NASH and in the mouse models of steatosis and NASH; Mfn2 re-expression ameliorates the disease in the NASH model mice, whereas, liver-specific deletion of Mfn2 induces inflammation, triglyceride accumulation, fibrosis, and liver cancer [143]. Moreover, reduced Mfn2 levels are also detected in mouse models of steatosis or NASH, and its re-expression in a NASH mouse model ameliorates the disease progression [143–145]. The inverse correlation between Mfn2 and fatty liver disease progression suggests that mitochondrial fusion is involved in the pathophysiology of fatty liver disorders. Mechanistically, Mfn2 specifically binds and extracts phosphatidylserine (PS) into the mitochondrial membrane domains and promotes mitochondrial phosphatidylethanolamine (PE) synthesis [143]. Consequently, hepatic Mfn2 deficiency reduces PS transfer and phospholipid biosynthesis, and promotes endoplasmic reticulum (ER) stress resulting in liver disease such as NASH and liver cancer; ablation of Mfn2 in the liver disrupts of ER-mitochondrial PS transfer [143]. The mice fed with a high-fat diet also show reduced expression of Opa1, but, this can be reversed by treatment with liraglutide, an anti-diabetic drug [114] Hepatic-specific ablation of Opa1 increases the risk of HFD-induced NAFLD [146]. Mechanistically, the proteolytic cleavage of L-Opa1 is increased in the liver by prohibitin-2 deficiency [146]. These molecular alterations are associated with lipid accumulation, decreased gluconeogenesis, and extensive liver damage. However, re-expression of L-Opa1 through adenovirus delivery restores the function of mitochondria in the hepatocytes and protects against NAFLD [146].

Re-activation of mitochondrial fusion protects against fatty liver disease. Exercise counteracts NASH by improving Mfn1 and Mfn2 expression, which promotes mitochondrial fusion and helps maintain mitochondrial homeostasis in the high-fat diet fed Sprague-Dawley rats [147]. This suggests that normalized mitochondrial fusion may preserve liver function under high-fat stress. Bitter gourd (BG) is a popular fruit in Asia with several medicinal properties. Bitter gourd intake increases Mfn1 expression, superoxide dismutase activity and mitochondrial respiratory function, and decreases sterol regulatory element binding protein/fatty acid synthase (SREBP-1/FAS) pathway activation [148]. Besides, salvianolic acid B is an anti-oxidant derived from *Salvia miltiorrhiza* that lowers ALT, AST, TG, and TC levels in high-fat diet fed mice [149]. The histological characteristics of inflammation and steatosis are also attenuated by salvianolic acid B by enhancing Mfn2-related mitochondrial fusion and reducing mitochondria-related hepatocyte death [149].

## Mitophagy

Autophagy is a mechanism through which eukaryotic cells recycle misfolded or aggregated proteins and damaged organelles such as the mitochondria through the lysosomes to generate amino acids, glucose, and fatty acids [150–154]. The macroautophagy process by which the mitochondria undergo degradation is called mitophagy [155, 156]. As shown in Figure 2, mitophagy plays a significant role in maintaining mitochondrial and cellular homeostasis during hepatic stress, and reduced mitophagy is associated with mitochondrial dysfunction and liver failure [157]. The best studied mechanism of mitophagy is the one mediated by PINK1 and Parkin proteins [74, 158–160]. In healthy mitochondria, PINK1 is degraded by the matrix processing peptidase (MPP) and the presenilin-associated rhomboid-like (PARL) protease [161]. When mitochondria are depolarized, stabilized PINK1 accumulates on the outer mitochondrial membrane, phosphorylates Mfn2 at Thr 11 and Ser 442, and recruits Parkin onto the OMM [162]. Alternatively, PINK1 promotes recruitment of Parkin to the OMM by phosphorylating ubiquitin and Parkin [20]. Cytosolic Parkin translocates to the OMM when deubiquitinated by USP8 [163]. BAG3 translocates to the OMM with Parkin, but, this process is prevented by physical interaction between Parkin and p53 [164]. Parkin ubiquitinates proteins on the OMM of depolarized mitochondria and promote the interaction of ubiquitinated OMM proteins with the mitophagy adaptor proteins such as p62, NBR1, and HDAC6, all of which contain an ubiquitin binding domain and a LC3-interacting region [165]. Finally, LC3 facilitates mitophagy when recruited by the adaptor proteins. Parkin-mediated ubiquitination of OMM proteins can be reversed by USP15 and USP30. PINK1 also promotes localization of LC3 receptors such as optineurin, NDP52, and TAXBP1, which recruit LC3 and facilitate mitophagy [166].

Several LC3 receptors are located on the mitochondria that can directly bind to LC3 and recruit damaged mitochondria to the autophagosomes. Proteins such as Nip3-like protein X (NIX) and BCL2/Adenovirus E1B 19 kDa Interacting Protein 3 (Bnip3) contain a BH3 domain that interacts with LC3 [167, 168]. NIX is required for the removal of mitochondria from reticulocytes during the process of maturation and form functional red blood cells [169]. Moreover, under hypoxic conditions, hypoxia-inducible factor-1α (HIF1α) increases NIX mRNA levels and the NIX protein is phosphorylated at Ser81 to mediate mitophagy [140]. Furthermore, NIX participates in Parkin-dependent mitophagy by recruiting NBR1 to the mitochondria and the knockdown of both Parkin and NIX synergistically reduces the levels of mitophagy [170, 171]. Homodimerization of Bnip3 is necessary for its interaction with LC3, which is further regulated by Ser17 and Ser24 phosphorylation near the LIR motif [172]. FUN14 domain-containing protein 1 (Fundc1) is a mitochondrial outer membrane protein that mediates hypoxia-induced Parkin-independent mitophagy in mammalian cells by binding to LC3; moreover, LC3 is regulated through the phosphorylation of Fundc1 at Ser17 by Unc-51 like autophagy activating kinase-1 (Ulk1) and Ser13 by casein kinase II or CK2 [14, 159, 173, 174].

Bnip3 expression is highly induced upon fasting in the liver of high-fat diet fed adult mice [175, 176]. Moreover, Bnip3 silencing increases lipid biosynthesis in the liver and is accompanied by elevated ATP levels, reduced AMP-regulated kinase (AMPK) activity, and increased expression of lipogenic enzymes [175, 176]. Conversely, in the liver of fasting Bnip3 null mice, β-oxidation of fatty acids and hepatic glucose output is reduced, and mechanistically linked to increased mitochondrial mass and hepatocellular respiration in the presence of glucose [177, 178]. The Bnip3 null mice liver mitochondria exhibit lower mitochondrial membrane potential, abnormal structure, and reduced oxygen consumption, and are associated with increased ROS, inflammation, and steatohepatitis-like features [177]. These results demonstrate that Bnip3-related mitophagy maintains mitochondrial integrity in the liver by decreasing mitochondrial mass, and plays a significant role in the regulation of lipid metabolism and liver disease. The role of Parkin-related mitophagy was first reported in alcoholic liver disease. Alcohol intake significantly elevated mitochondrial damage and oxidative stress in the livers of Parkin-knockout (KO) mice compared to the wild type [179]. Besides, the liver mitochondria of Parkin KO mice were swollen and lacked cristae compared to normal mitochondrial structure observed in the livers of WT mice [179]. These findings were subsequently confirmed in the high-fat diet fed NAFLD model mice, wherein, the expression of PINK1 and Parkin was significantly downregulated and associated with activation of the mitochondria-related apoptotic pathway and mPTP opening [147]. Metformin suppressed p53-mediated hepatocyte apoptosis in response to HFD through Parkin-induced mitophagy [180]. Acyl-CoA lysocardiolipin acyltransferase-1 (ALCAT1) catalyzes cardiolipin remodeling, which is involved in the pathology of several aging-related diseases [181]. Mitophagy arrest and mitochondrial dysfunction in NAFLD is reversed by the targeted deletion of ALCAT1 [181]. ALCAT1 deficiency also improves mitochondrial architecture, mitochondrial DNA (mtDNA) fidelity, oxidative phosphorylation, and other pathological changes induced by NAFLD [181]. These results suggest that mitophagy protects against NAFLD/NASH. Furthermore, mitochondrial fission is important for mitophagy induction in the liver hepatocytes and other cell types [135, 182]. However, excessive mitochondrial fission triggers hepatocyte death by inducing mitochondrial damage. Therefore, the relationship between mitochondrial fission and mitophagy in NAFLD needs to be further investigated. Recent study shows that exposure to alcohol increases mitochondrial fission and inhibits mitophagy, thereby suggesting that mitophagy is independent of mitochondrial fission in the alcoholic liver disease [183]. Moreover, in the renal ischemia reperfusion injury model, mitophagy is repressed and mitochondrial fission is activated, resulting in mitochondrial fragmentation [159, 184], Interestingly, induction of mitophagy removes damaged mitochondria and blocks mitochondrial fission [109]. This suggests that mitophagy corrects excessive mitochondrial fission, but, this concept needs to be further validated in the NAFLD or NASH model.

## Conclusion and Future Perspectives

Oxidation of fatty acids in the mitochondria is one of the main sources of energy for eukaryotic cells. Dysregulated fatty acid oxidation (FAO) in the mitochondria contributes to fat accumulation and hepatic steatosis. Mitochondria are dynamic organelles that continuously undergo fusion and fission. Fusion involves joining of the inner and outer mitochondrial membranes of two mitochondria, whereas, fission generates two metabolically distinct daughter mitochondria that may either maintain or lose their membrane potential. Excessive mitochondrial fission is an early event that triggers hepatocyte death by inducing mitochondrial dysfunction. In response to mitochondrial damage, depolarized mitochondria are targeted for degradation by autophagy (mitophagy). If mitochondrial damage is too severe to be fixed by the intracellular protective mechanisms, the dysfunctional organelles break open and release several activated apoptotic factors that trigger programmed cell death. Therefore, the dynamic nature of the double-membrane-bound organelles as well as mitophagy is critical for maintaining their function, structure, and the inheritance of mitochondrial DNA. Taken together, reduced levels of β-oxidation and increased lipogenesis results in lipid accumulation in the hepatocytes that generates excessive ROS and hepatocyte injury that further drives hepatic inflammation and fibrosis. The mitochondrial quality control mechanisms restore mitochondrial function and prevent the progression of NAFLD.

The mitochondrial unfolded protein response (UPR^mt^) pathway represents a retrograde mitochondria-to-nucleus signaling pathway induced by mitochondrial proteotoxic stress [185]. This pathway was first identified in the late 1990s, and is an area of considerable scientific research using the worm and mammalian models of human aging-related degenerative diseases and metabolic disorders [186, 187]. The molecular mechanisms underlying UPR^mt^ in the progression of NAFLD are not well understood and needs further investigation.
